# A novel decentralized federated learning approach to train on globally distributed, poor quality, and protected private medical data

**DOI:** 10.1038/s41598-022-12833-x

**Published:** 2022-05-25

**Authors:** T. V. Nguyen, M. A. Dakka, S. M. Diakiw, M. D. VerMilyea, M. Perugini, J. M. M. Hall, D. Perugini

**Affiliations:** 1 Presagen, Adelaide, SA 5000 Australia; 2 School of Computing and Information Technology, University of Wollongong, Wollongong, NSW 2522 Australia; 3 School of Mathematical Sciences, The University of Adelaide, Adelaide, SA 5005 Australia; 4 Ovation Fertility, Austin, TX 78731 USA; 5 Texas Fertility Center, Austin, TX 78731 USA; 6 Adelaide Medical School, The University of Adelaide, Adelaide, SA 5000 Australia; 7 Australian Research Council Centre of Excellence for Nanoscale BioPhotonics, Adelaide, SA 5005 Australia; 8 School of Physical Sciences, The University of Adelaide, Adelaide, SA 5005 Australia

**Keywords:** Computer science, Embryology, Software, Medical imaging

## Abstract

Training on multiple diverse data sources is critical to ensure unbiased and generalizable AI. In healthcare, data privacy laws prohibit data from being moved outside the country of origin, preventing global medical datasets being centralized for AI training. Data-centric, cross-silo federated learning represents a pathway forward for training on distributed medical datasets. Existing approaches typically require updates to a training model to be transferred to a central server, potentially breaching data privacy laws unless the updates are sufficiently disguised or abstracted to prevent reconstruction of the dataset. Here we present a completely decentralized federated learning approach, using knowledge distillation, ensuring data privacy and protection. Each node operates independently without needing to access external data. AI accuracy using this approach is found to be comparable to centralized training, and when nodes comprise poor-quality data, which is common in healthcare, AI accuracy can exceed the performance of traditional centralized training.

## Introduction

Bias in AI, and its subsequent limitations of scalability, are starting to emerge as common themes in the AI healthcare sector. It has been recently proposed that these limitations are a consequence of training on ‘narrow’ datasets that do not represent real-world clinical or patient diversity^1,2^. Data diversity, and using data from multiple sources, have demonstrated greater potential to train AI that is more accurate and generalizable compared with AI trained on a larger (less diverse) dataset from a single source^3–8^.

In healthcare, access to these diverse datasets can be challenging. Not only are medical data distributed across many institutions globally, but centralized aggregation of data for AI training is increasingly restricted due to legal and regulatory barriers that prevent movement of data outside of the region of origin, in order to protect data privacy^9,10^.

Data quality can also pose a challenge if there is no way to assess quality of individual datasets that are distributed. For many real-world problems, data can be inherently poor-quality due to uncertainty, subjectivity, errors, or subjected to adversarial attack^11–13^. This problem is exaggerated when private data at each locality cannot be manually seen or verified. Therefore, minimizing the negative impact of poor-quality data on AI performance is paramount, and the ability of any approach to handle realistic levels of data noise will represent a core part of its scalability.

This study assesses the efficacy of the decentralized AI training approach, firstly on a non-medical dataset with synthetic data noise, and secondly on a medical dataset, to measure generalizability across multiple locations. We also employ methods for optimizing topologies of a Pattern-based framework that allows a trade-off between accuracy and cost to be specified. Importantly, we show that the accuracy of AI resulting from our approach is comparable to a scenario where all data are centralized. Furthermore, when nodes contain poor-quality data, which is common in real-world scenarios, the accuracy of the AI can exceed traditional centralized training. We conclude that decentralized AI training can be made both *practical* and *scalable* to within a desired tolerance of generalizability, all whilst protecting data privacy.

This article is organized as follows. After summarizing related works in “Related works”. below, the Results are presented in second section. The experiments are divided into those that consider a non-medical dataset (“Non-medical dataset”), including scenarios labeled i. through iv., and those that consider a medical dataset (“Medical dataset”). The “Discussion” is presented in third section. Lastly, the “Methods” are described in fourth section, including the experiment design, training procedure, and the composition of the non-medical and medical datasets as “Experiment design and training procedure”, “Non-medical dataset composition” and Medical dataset composition, respectively.

### Related works

One recent development that addresses the challenges associated with training AI using distributed and private datasets is federated learning^14,15^. Federated learning encompasses any machine learning approach where clients (such as devices or data centers) with access to their own local datasets collaborate to solve a problem without exchanging the data in raw format, coordinated by a central service^16^. The field of federated learning has rapidly expanded into the area of healthcare^17–21^, in medical applications in particular^22–25^ bringing a wide range of methods for AI training across distributed devices or data silos (horizontal or sample-based federated learning), data centers within an organization with potentially overlapping records (vertical or feature-based federated learning)^26^, and datasets that are not ‘independent and identically distributed’ (IID)^27,28^.

Some federated learning techniques need heavy encryption to allow aspects of potentially sensitive training parameters to be shared to a central server for training^17,29–31^, which can be computationally expensive, placing constraints on the practicality and scalability of the technique. In applications such as healthcare, private medical data cannot be legally shared at all, and a completely decentralized and data privacy preserving approach is required^16,28^. Additionally, federated learning typically relies on batch-by-batch updates to a model from clients, which can be difficult to scale to a high number of data centers due to the high network costs, even when relying on Pattern-based transfer reduction frameworks, such as Ring Reduce^32^ or Ring Allreduce^33^.

The objective of this study was to create a completely decentralized, data-centric, cross-silo AI training algorithm that does not require batch-by-batch updates to a model on a central server, and can achieve high accuracy for low network costs, even on non-IID datasets. In this paper we unveil a novel, data agnostic implementation of a robust Decentralized AI Training Algorithm (DAITA)). We combine several techniques such as federated learning^15^, knowledge distillation^34^, and a scalable Pattern-based or Directed Acyclic Graph (DAG) framework. Our algorithm implements a cost-effective simplification of full distributed training, checks for security violations, and uses weight averaging to prevent reconstruction of any data.

## Results

First, we considered a non-medical, cat and dog image dataset^35^, where ground-truth outcomes were definitively known, and synthetic noisy data were injected into the dataset to simulate real-world unbalanced distributions of data and poor-quality data scenarios. Different node and cluster configurations were implemented. Given the best experimental setting for this non-medical dataset, the technique was then applied to an embryo dataset obtained from multiple IVF clinics to test the performance in a real-world scenario.

### Non-medical dataset

**Figure 1:**
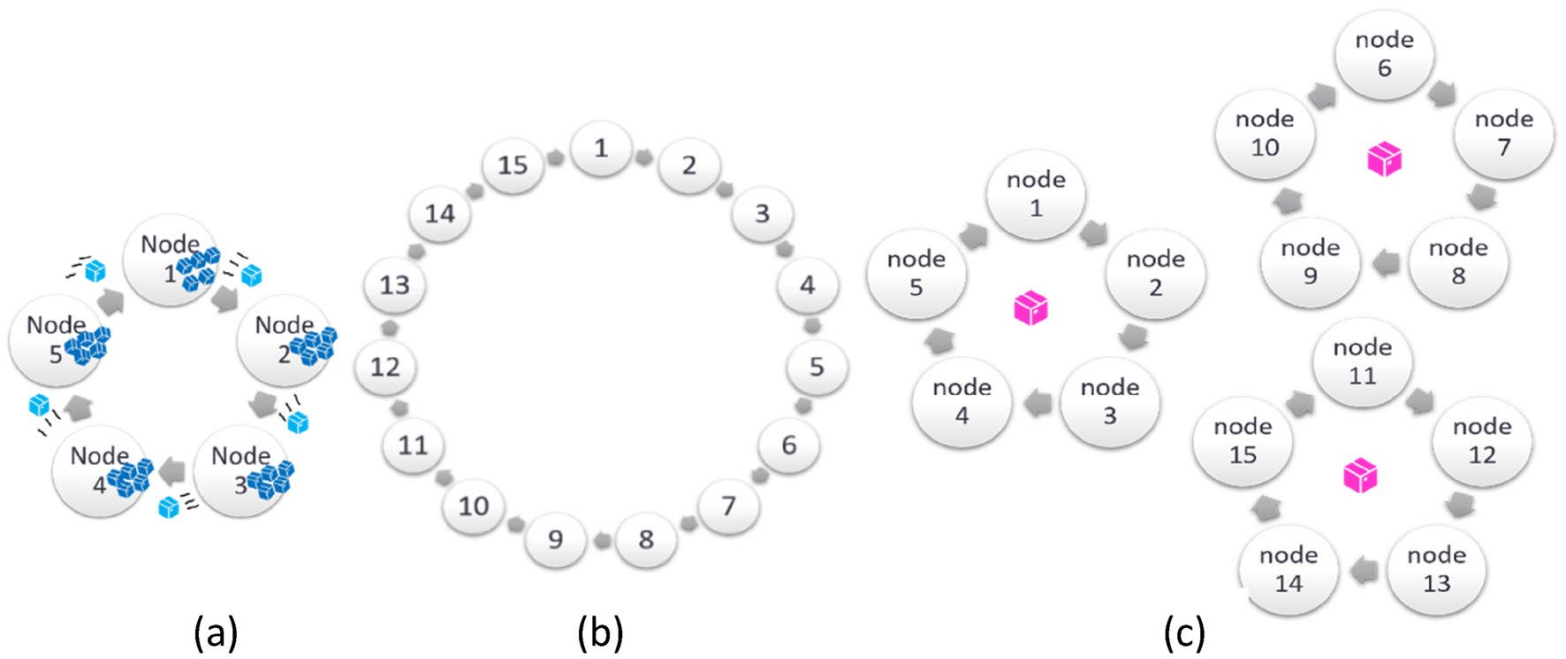
Illustrations of 5-node (**a**), 15-node (**b**) with single cluster scenarios, and 5-node each in 3-cluster scenario (**c**).

The source and composition of the non-medical dataset is described in “Training procedure” in the “Methods” section. The configuration of distributed nodes (data sources) and clusters (groups of nodes) used in the experiments are shown in Fig. 1. Below the key results of three decentralized training scenarios are summarized.

#### Experiments comparing cleansed and noisy datasets

In these experiments, a 5-node, 1-cluster setting as illustrated in Fig. 1a was used together with its “Training procedure” described in the “Methods” section. A model ${\mathbb{M}}^{1}$ was trained using a transfer dataset via distillation and using the plurality of all trained Generalists as its teacher models. The final model and weights were obtained based on the epoch reporting the highest balanced accuracy on the validation set.

A second model ${\mathbb{M}}^{2}$ was created by the ensemble of all trained Generalist models without the use of knowledge distillation. This process can occur on a separate server where there is no restriction in downloading the transfer dataset to the local machine. These two models’ results are compared with the baseline results, which represent traditional training on all the data centralized on one node.

Results shown in Table 1 confirm the decentralized training algorithm performs well compared with the centralized baseline results. When using a cleansed dataset, there was minimal difference reported in the accuracy between decentralized training (${\mathbb{M}}^{1}$ and ${\mathbb{M}}^{2}$) and the centralized baseline results.

**Table 1:** Model result comparison using 5 evaluation metrics: mean accuracy, class 0 (cat) accuracy, class 1 (dog) accuracy, balanced accuracy, and log loss.

Models	Total accuracy	Class 0 (Cat)	Class 1 (Dog)	Balanced accuracy	Log loss
**Cleansed data**					
Baseline	98.44	98.62	98.26	98.44	0.061
${\mathbb{M}}^{1}$	98.42	98.49	98.35	98.42	0.045
${\mathbb{M}}^{2}$	98.62	98.13	99.11	98.62	0.038
**Noisy data**					
Baseline	75.31	57.34	93.32	75.33	1.235
${\mathbb{M}}^{1}$	78.00	57.68	98.35	78.01	0.421
${\mathbb{M}}^{2}$	73.78	48.49	99.11	73.80	0.598

When using a noisy dataset, Table 1 shows that the decentralized training algorithm (${\mathbb{M}}^{1}$) performs better (+ 2.7% accuracy) than the centralized baseline. The experiment was repeated multiple times using different dataset configurations, and similar improved accuracy was achieved using decentralized training. This result was unexpected and significant in demonstrating the utility of the DAITA for data privacy, performance (accuracy and generalizability), and ability to robustly train in the presence of noisy (poor-quality) data. Noisy data are likely to occur in most real-world situations, particularly in a decentralized situation where there are multiple data owners and limited data transparency. Nevertheless, all the local Specialist models showed reduced generalizability compared with the Baseline model since they have access to much smaller sets of training data than the baseline training set.

Table 1 also shows that the model trained without using knowledge distillation (${\mathbb{M}}^{2}$) performed worse (− 1.5% accuracy) than the baseline because the ensemble of individual Generalists emerges locally at each node. Therefore, this extra step of creating an ensemble will be disregarded while the use of knowledge distillation will be emphasized for now, to simplify the experimental design, while more options of a transfer set were examined.

Since the experimental results for the cleansed training set is similar to the baseline results and close to the maximum 100% accuracy, in the following sections, all experiments were conducted only on the noisy training-validation datasets, with a lower 75% baseline accuracy, to better assess variations between different decentralized training approaches. While the total accuracy and balanced accuracy are similar in value, as shown in the bottom half of Table 1, the final model trained on the transfer set shows its superiority to the baseline results when knowledge distillation is used (the class 1 accuracies exceed those of class 0 due to the unbalanced class distribution and the uneven amount of noise synthetized to each class).

#### Experiments comparing choices of transfer dataset

**Figure 2:**
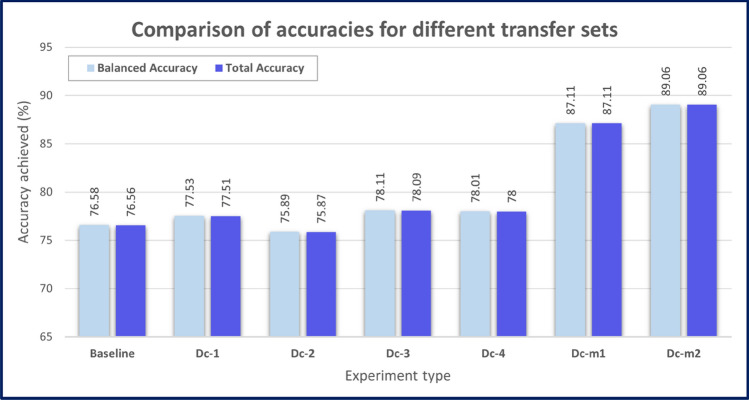
Comparison of decentralized model results for different transfer set scenarios. Baseline indicates an experiment where all data are centralized, and training occurs on this central node. Dc-1 to Dc-4 refer to experiments where individual nodes (1–4) are chosen as the only transfer set. Dc-m1 indicates a scenario where decentralized training occurs, but the transfer set is the theoretical centralized set of all data. Dc-m2 indicates a scenario where decentralized training is followed by a final process whereby all final models are distilled together at each node in term, with one full traversal of all nodes.

The algorithm in “Experiments comparing cleansed and noisy datasets” requires a separate transfer set, however in practice, a separate transfer set might not be available. In that case, the existing data at each node can play a role as the transfer set. In this section, we empirically investigate various options for the choice of transfer set. Figure 2 compares the results of the following experiments:

1. Dc-i: after proceeding through the training on 5 nodes’ data using the DAG topology shown in Fig. 1a, and using multiple Teacher knowledge distillation, ${\mathbb{M}}^{1}$ is ultimately trained at its final step with a single node’s data (i-th node’s data), as the transfer set for ${\mathbb{M}}^{1}$.
2. Dc-m1: Represents the optimistic scenario where all the nodes’ data can be gathered collectively and Dc-m1 is the result when ${\mathbb{M}}^{1}$ is trained on this collective transfer set.
3. Dc-m2: Represents the realistic data privacy preserving scenario, where multiple transfer sets are utilized. ${\mathbb{M}}^{1}$ will travel to each node and, in turn, take each node’s data as its transfer set. The final model is trained on the local data (seen as a local transfer set) and consulting the knowledge from the plurality of trained Generalist models. Since the process requires extensive data transfer, the final model and all trained Students are transferred to each node for only one round (see Fig. 1a).

Note that the intention of Fig. 2 is specifically to compare the performance in a given decentralized scenario, for different choices of transfer set. Note that a shared validation set was used for all decentralized models, and this set was identical to that used in the baseline training. The validation set was placed on a single node where all the decentralized models could eventually be moved in order to report on the validation set.

In experiments Dc-1–4, the transfer set was chosen to be a dataset on one of the nodes only. This is compared to a scenario in experiment Dc-m1 where a theoretical transfer set consists of all centralized data. In contrast, a privacy-preserving decentralized approach is conducted in experiment Dc-m2, where each final model at each node is sent to every other node, to be distilled on each node’s data as a transfer set, thus using combined data as a transfer set without transferring the private data from any node.

Figure 2 demonstrates that the performance of almost all the decentralized AI models outperform the baseline result. Even if the transfer set is as small as a single node’s data, the results of experiments Dc-1 to Dc-4 are still similar to the baseline result. In terms of using multiple transfer sets, both experiments Dc-m1 and Dc-m2 show a significant improvement in accuracy (by 9% and 11%, respectively) compared with the baseline results. This suggests that a combination strategy would be beneficial for the transfer set. In fact, the model created for experiment Dc-m2 exceeds the performance of the model for Dc-m1, where data are centralized. This is due to Dc-m2 having: (a) an order of traversal through the nodes (a Pattern-based DAG approach); and (b) a new hyperparameter corresponding to total number of epochs on each node, which can be tuned to achieve optimal results.

Since experiment Dc-m2 empirically was considered to be the most robust and feasible methodology of using data as the transfer set, it was used in the following experiments.

#### Experiments comparing scalability using clustering and varying epochs at each node

**Figure 3:**
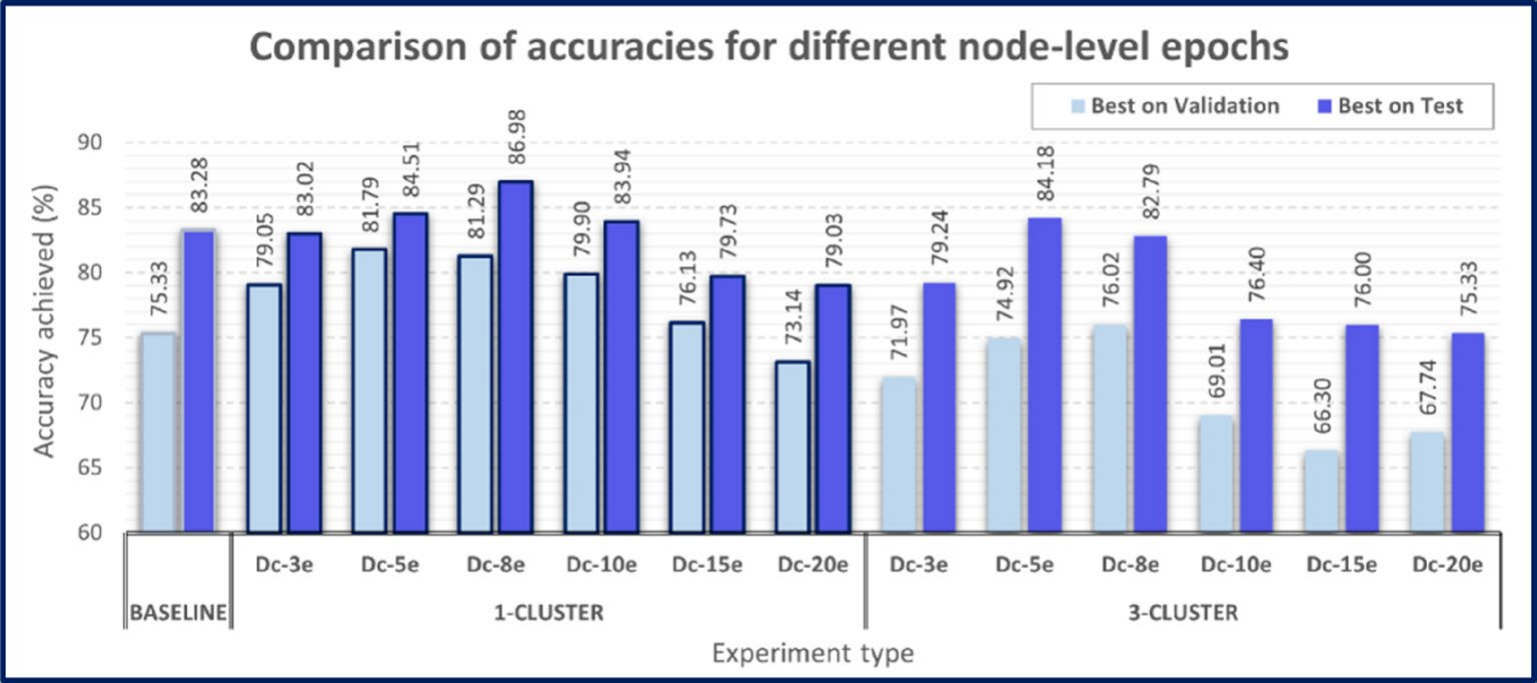
Comparison of 15-node decentralized experiments where the number of node-level epochs for each node are altered and compared. For all node-level training of k epochs before transferring to neighboring nodes, the experiment is denoted Dc-ke. A clustering scenario where 15-nodes are split into 3 clusters of 5 nodes each, are compared to the results from a full ring of 15 nodes.

To test the scalability of the DAITA, a 15-node scenario was explored. Two types of DAG topologies are considered, namely 1-cluster (refer Fig. 1b) and 3-cluster (refer Fig. 1c) with even node distributions. The decentralized models will be trained using these two clustering arrangements.

We specifically tested the influence of the number of epochs at each node on the decentralized AI model performance. For each topology, the final decentralized models were trained using 3 to 20 epochs, and the corresponding accuracy results are denoted as Dc-$i$ e where $i\in \{\mathrm{3,5},\mathrm{8,10,15,20}\}$. For instance, Dc-3e denotes the approach where the final decentralized model is sent around to each node once and then trained locally with 3 epochs. All results reported are on the test set. The term “Best on Validation” in Fig. 3 is used to denote results of models that were selected based on the best balanced accuracy on the validation set. The term “Best on Test” is used to denote results that are selected on the best balanced accuracy on the test set. The “Best on Test” results are reported purely for the assessment purpose of the model’s best predictive capability.

Node clustering, otherwise described as the 3-cluster scenario, was used to improve the scalability of the decentralized AI technique. The decentralized AI training was carried out within each individual cluster of nodes concurrently, then further training occurred between clusters, in a hierarchical way, where each cluster represents a single node. This approach defines the DAG, improves load-balancing, reduces the number of trained Generalist models that need to be transferred between nodes, and hence improves data transfer efficiency and cost, and overall training time.

#### Experiments comparing data transfer efficiency

The poor performance from node clustering is primarily due to cluster-wide Generalist models only visiting each nodes’ data once within a given cluster as can be seen in Fig. 3 for 3-cluster scenario. Even though the final decentralized model ${\mathbb{M}}^{c}$ has access to all data across all clusters, a single visit to each node is not sufficient to appropriately train ${\mathbb{M}}^{c}$. The node’s data size is important; however, the results shown in Fig. 3 indicate that clustering configuration is a significant factor contributing to the drop in accuracy. Since the clustering is necessary to ensure scalability in a real-world situation, a larger number of clusters may further reduce the accuracy of ${\mathbb{M}}^{c}$. The following experiments will confirm that when ${\mathbb{M}}^{c}$ travels to each node within clusters more than once, its accuracy and generalization can increase to a level comparable with the baseline results.

Results in Table 2 show that when ${\mathbb{M}}^{c}$ visits each node at least three times, the final model accuracy improves. Importantly, ${\mathbb{M}}^{c}$’s accuracy can exceed the baseline accuracy results by approximate 3% on average. The scenarios in Table 2 are denoted Dc-1e-5t and Dc-2e-5t, which represents ${\mathbb{M}}^{c}$ visiting each node 5 times and the number of epochs at each node being 1 or 2, respectively. Another observation that is not shown in Fig. 3 but can be seen here is that the per-class “Best on Validation” accuracies of the decentralized AI models are much more balanced than the baseline results, accounting for at least 14% improvement in accuracy for Class 0. The decentralized training technique and the knowledge-based distillation integration has shown to some extent, an ability to accommodate the unbalanced class distribution in this case.

**Table 2:** Trade-off between data transfer and model accuracy.

Exp-id	Description	Total Acc	Class 0	Class 1	Balanced Acc
**Best on validation**					
Baseline		75.31	57.34	93.32	75.33
Dc-1e-5t	1 epoch at each node	76.98	75.01	78.95	76.98
Dc-2e-5t	2 epochs at each node	79.91	71.01	88.83	79.92
**Best on test**					
Baseline		83.27	71.06	95.5	83.28
Dc-1e-5t	1 epoch at each node	85.04	73.5	96.61	85.06
Dc-2e-5t	2 epochs at each node	87.75	90.14	85.36	87.75

There exists a trade-off between network transfer cost and the final AI model’s accuracy. Empirically, the final model exhibits higher performance when it is given a sufficient number of training epochs to learn from data at each node. As a result, a Pattern-based DAG approach with a tunable number of epochs before transfer of the Generalist model to another node effectively abstracts the problem of optimizing network transfer costs against accuracy to a hyperparameter search, thus allowing for specifying a desired threshold of accuracy for a given problem, whilst retaining scalability.

Consider a worked example where there are 5 nodes arranged in a ring for our decentralized training approach, as per Fig. 1a, resulting in a model ${\mathbb{M}}^{c}$. As a comparison, consider a 4-worker and 1-master client–server architecture for traditional distributed training, resulting in a model ${\mathbb{M}}^{d}$. Assume each model is trained for 100 epochs with batch size 16 on the dataset of 4,500 images.

Using our decentralized approach, let us propose that ${\mathbb{M}}^{c}$ will be trained with 5 nodes’ data of even size (900 images), using 5 Teacher models and knowledge distillation. While training, ${\mathbb{M}}^{\mathrm{c}}$ is assumed to move to each node, along with the 5 Teacher models, and is trained for 2 epochs using local data before being moved to the next node. Since each model needs to be transferred to the node’s local storage, a model weight transfer operation needs to be performed each time. If the model ${\mathbb{M}}^{\mathrm{c}}$ traverses the entire 5-node topology for 10 rounds, each Teacher model on each node trains for 2 epochs * 5 nodes (each Teacher model trains on each node) * 10 rounds = 100 total epochs. This requires 5 nodes * 10 rounds * (5 Teacher models + 1 final ${\mathbb{M}}^{\mathrm{c}}$ model) = **300** model weight transfer operations.

In the case of full distributed training, assume ${\mathbb{M}}^{\mathrm{d}}$ trains on 1,125 images allocated at each of the 4 worker nodes, where the master node has no data, and acts as an orchestrator. For a distributed training run, an epoch would contain 1125/16 ≈ 70.3 batches of size 16. The number of model’s weight transferred for a single batch is 4 workers * 2 times (back and forth) = 8 per-batch transfer operations between 4 workers and the 1 master node. If it is assumed that ${\mathbb{M}}^{\mathrm{d}}$ is trained with 100 epochs, the total number of times the network’s weight being transferred would be 70.3 batches * 8 per-batch transfer operations * 100 epochs = 56,240 model weight transfer operations. Hence, by using the decentralized training, while the accuracy is maintained at a comparable level, the average number of transfers reduces by 187.5 times which is a reduction from 56,000 down to 300 transfers. The number of data transfer scales linearly with the number of nodes involved. The proposed knowledge-based decentralized training algorithm helps optimize the amount of data transfer, and ultimately minimize the data transfer costs especially when the decentralized AI training is scaled with many nodes.

Figure 3 (1-cluster) results show that the decentralized models’ accuracy can outperform the baseline, particularly when the final model is trained with 5 or 8 epochs at each node, which accounts for up to 15% improvement in accuracy. An interesting observation is when the final model remains at each node for longer (i.e. a greater number of epochs), the test set accuracy becomes *worse* than the baseline accuracy. This is because the decentralized model is prone to overfitting the local node’s data and ‘forgets’ what it learned in previously traversed nodes.

Figure 3 (3-cluster) results show that the decentralized AI models’ accuracy dropped by approximately 10% compared with the corresponding decentralized AI model using 1-cluster setting. The final models are also less accurate than the baseline results. As with 1-cluster, when the final model is trained with more epochs at each node, the test set accuracy worsens, though the expected network transfer costs decrease.

Nonetheless, the poor performance for this configuration is to be expected and effectively measures the extent to which clustering of nodes impacts the final decentralized model’s generalizability and performance. In the “Methods” section we describe a technique to address poor performance from node clustering, by optimizing data transfer costs against model accuracy.

### Medical dataset

In considering a medical dataset, we focused on the problem of assessing the viability of embryos in the IVF sector, using an existing algorithm called Life Whisperer Viability—a commercial in-market ML application for embryo selection^7^. A viable embryo is defined as one that leads to a clinical pregnancy for the IVF patient once transferred, and a non-viable embryo is considered to be one that does not lead to a clinical pregnancy. Images of embryos were collected from multiple clinics. The description of this medical dataset is shown in Table 4 and Fig. 6 in the “Methods” section under Medical dataset composition.

**Figure 4:**
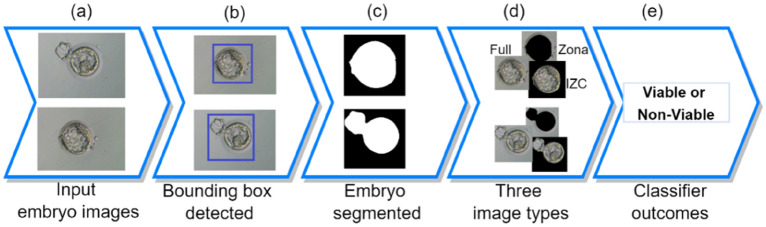
The workflow of predicting/identifying the viability of an embryo image.

Figure 4 presents the workflow or the process of predicting or identifying a given input embryo viable or non-viable. The process can be described briefly as follows. There are pre-processing and classifying stages. In the pre-processing stage, the trained detection model was utilized to detect the input embryo (a), and the results are represented as bounding boxes (b). The images were then cropped before feeding into another segmentation model which was trained with embryo image’s mask (c) and ultimately the process produces two more images (*zona pellucida* (Zona)-segmented and *intra-zonal cavity* (IZC)-segmented images) in addition to the cropped (Full) image (d). In the classifying stage, these three types of images were used as input to the classification model which is called AI model (i.e., an ${\mathbb{M}}$ model in the decentralized training). This classifier model would play the central role in predicting the Viable or Non-Viable outcomes of the input embryo images.

A 5-node 1-cluster configuration was used, where each node contained data from different clinics. A number of model training runs were conducted with different options of model architectures, parameters in which the 3-level loss function’s variables were considered an addition to AI model’s tunable hyper-parameters (see Supplementary Information S1). For the centralized training, the two weighting levels, namely sample and class weighting, can be deployed while for the decentralized model, all three-level weighting would be applicable.

The best AI models were selected based on the best log-loss value on the validation set, which represents a key selection metric that indicates generalizability in a more robust manner than balanced accuracy, for medical datasets.

The results of the cleansed and noisy test sets were then obtained and compared between the baseline centralized and decentralized AI (${\mathbb{M}}^{c}$) models.

Table 3 presents the per-class and total accuracy of the baseline and decentralized AI models for the embryo dataset. Their results are very comparable with slight shifts between the per-class accuracies. ${\mathbb{M}}^{c}$ gave slightly superior results in terms of total accuracy, with approximately 2% greater prediction accuracy for viable embryos compared to the baseline’ results.

**Table 3:** Comparison of model results on a medical (Embryo Viability) dataset.

Models	Non-viable	Viable	Total accuracy
**Cleansed data**			
Baseline	59.92	82.96	76.09
${\mathbb{M}}^{c}$	57.72	84.37	76.41
**Noisy data**			
Baseline	35.97	78.67	60.24
${\mathbb{M}}^{c}$	34.04	80.58	60.50

**Figure 5:**
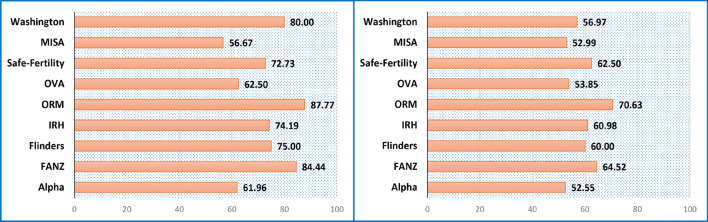
The decentralized model’s accuracy performance for individual clinic data in the cleansed test set (left graph) and in the noisy blind test set (right graph).

Figure 5 shows the results of ${\mathbb{M}}^{c}$ model for individual clinical centers’ data allocated in the cleansed test set (on the left) and in the noisy blind test set (on the right). The accuracies fall with a range from 56.67% to 87.77% for clinics’ data in the test set and from 52.55% to 70.63% for clinics’ data in the noisy blind test set. One clinical dataset (MISA) that performed the worst on the cleansed dataset is smallest set overall and hence a non-representative dataset (accounting for 3% of the test set). The accuracies across different clinics are nevertheless quite consistent overall.

## Discussion

Training generalizable, unbiased AI models using real-world diverse medical datasets that are distributed, private and poor-quality poses significant challenges in terms of accuracy, cost and scalability, particularly in highly regulated markets like healthcare.

The DAITA implementation incorporates knowledge distillation, enabling scalable model training to be executed at a much lower cost compared with distributed training, because it is achievable without the network traffic and server costs associated with a batch-by-batch gradient gathering procedure. The DAITA can furthermore be organized into a Pattern-based or DAG structure, lending itself to automation and cost/accuracy optimization.

Surprisingly, the final performance of the model, depending on the configuration chosen, can even *exceed* the baseline accuracy associated with training on a centralized dataset in the traditional manner. This accuracy uplift provides greater flexibility and choice in transition models from node-to-node, and hyperparameters can be tuned so that the decentralized training process can be treated as an optimization problem. In a case study of non-medical images, for a binary classification problem with a known level of mislabeling, AI model performance was shown to increase up to 11% above the baseline accuracy.

The reported uplift in performance on noisy datasets can be understood as originating in the additional stabilizing capability of federated learning methods, such as distillation training across data sub-divided across multiple nodes. Each Student model, trained with inputs distilled from multiple Teacher models across each node, combines the knowledge from individual biases derived from each node in order to stabilize the model training, and thus naturally gains an advantage over simply training a single model on a centralized dataset.

A medical image dataset, focusing on the binary classification problem of Embryo Viability, was examined. A range of separate clinics with different work practices, and different levels of noise and image quality were considered, encompassing many of the challenges encountered in a real-world scenario that would ordinarily prevent a robust AI model from being obtained. By adopting a DAITA strategy and optimizing for the total number of node-level epochs, with a three-level weighting—sample, class, and node-level, up to 2% performance increase compared with the centralized baseline was observed.

A comparison with state-of-the-art deep learning results in medical imaging, including prostate MRI segmentation^21^, and breast mammography^22^, shows a consistent trend federated approach can significantly improve upon the performance of models trained only at their local node, and report results comparable with that of a centralized dataset^39^.

Note that, while using a cleansed dataset led to minimal difference in accuracy between decentralized training and the baseline, using a noisy dataset led to much larger difference between the two. We found that DAITA, when equipped with a novel loss function (see Supplementary Information S1) and multiple Teacher models for distillation, results in an uplift in accuracy similar to that of data cleansing techniques^39,40^.

A further optimization strategy can also be adopted, where the total number of model transfers per node-level epoch can be reduced, either by reducing the hyperparameter associated with the number of node-level epochs, or by treating the DAG of nodes differently, using *clustering*, and only transferring representative models between the clusters, rather than the nodes. Clustering drastically reduces the total number of model transfers required, albeit at the expense of accuracy improvement above the baseline result.

We note that tackling data privacy issues and localized datasets will become increasingly more important, as ML techniques expand to increasingly complex real-world datasets in healthcare and other industries that involve sensitive data, with the demands that they generalize correctly on diverse datasets with different distributions, without violating privacy.

## Methods

### Experiment design and training procedure

In training on distributed datasets, a strategy must be chosen, in how the workload will be divided among the compute nodes. In one method, Data Parallelism, the dataset is split into partitions. Between two forms of Data Parallelism, namely Fully Distributed Training and ‘Pattern’ (or DAG based Training), as described in Supplementary Information S1, we show that the Pattern method exhibits superior scalability and cost-effectiveness^15–17^. Combining the Pattern method with distillation can further improve the efficiency of the training algorithm, in a way that allows an ML engineer to optimize a solution, either for cost, or accuracy.

In this article, we consider a *n*-node ring problem, where each of the *n* nodes individually suffers from a small-data problem. We successfully train a high-performing, generalizable model on *the n* nodes. Further, we explore a novel Clustering algorithm, by which model transfer costs (that scale quadratically as the number of nodes increases) can be further reduced by limiting the nodes to which Teacher models are transferred, to within a Cluster. This alternative topology simplifies the *n*-node ring into *m* separate Clusters of rings, where each ring can contain a different number of nodes if desired, and where each Cluster is used to produce a representative model. From this point on, the Clusters are treated for all intents and purposes as nodes. For example, in the case of 15-node ring, with 3 Clusters of 5-nodes each, this limits the total model transfers from ${15}^{2}=225$, to $3\times {5}^{2}+{3}^{2}=84$ transfers per circuit of the nodes. For more information, see Supplementary Information online S1.

#### Decentralized training and knowledge distillation

Distillation is a powerful method that uses a trained Teacher/Specialist model to guide the training of a Student/Generalist model, without directly requiring expensive model weight updates to be transferred across nodes for every batch^34^. This is achieved by allowing a Teacher model to compute its predicted outputs (probabilities and losses) at the same time as the Student model is training on a node, on the node’s own local dataset (called a transfer set), and to contribute to the loss function of the Student model as it is training. The Teacher outputs (or soft labels) are compared to the Student model outputs via a divergence function such as the Kullback–Leibler (KL)-Divergence^36^, which compares the relative ‘distance’ between the two models’ output distributions and adds to the loss function being used to train, such as the standard cross-entropy loss. Multiple Teacher models can be used to assist a Student model at the same time, with difference weightings, and they do not have to be the same kind of neural network architecture, making it a powerful and general approach. Additional details regarding the specifics of the loss functions used, and pseudocode for the training Algorithm, can be found in the Supplementary Information S1.

We therefore are able to cast the problem of decentralized training as simply an optimization problem, where we now include additional hyperparameters for Student–Teacher weighting (i.e. the temperature and *alpha* parameter, which controls how much the training is biased toward the Teacher model input versus the Student model training)^36^, and the Pattern/DAG parameters such as the number of epochs for each Student to reside before being transferred to another node, and how many ‘rounds’ across all nodes to compute. The performance of a final distilled model can be assessed on a given transfer dataset.

As a final step, we consider a final ‘closing’ process that exhibits superior generalizability. After training *n* Student models in parallel across a topology (for *n* nodes), the final *n* models are distilled together at each node, treating the local dataset of each node as a transfer dataset, for *k* epochs (at the node level), before transferring all *n* models to a neighboring node, repeating at least one full cycle of the nodes. This final process is more network-transfer intensive, but essentially treats the entire distributed dataset as a transfer dataset, rather than using a single node’s dataset as the transfer dataset, thereby achieving more balanced performance.

The model architectures used in the experiments presented in this work include ResNet18^37^, ResNet50 and DenseNet121^38^ with the pre-trained model using the ImageNet dataset. The network parameters are selected by running multiple runs using the baseline cleansed dataset. The optimal values for hyperparameters such as learning rate, regularization methods, weight decay, loss function, or batch size, etc., were identified and then used throughout all the experiments for decentralized training.

For each architecture considered above, the network weights in the feature space were obtained from a mode pre-trained on ImageNet, with network surgery performed to add a fully-connected layer with a binary output (cat/dog, or non-viable/viable, for the non-medical and medical datasets, respectively). A softmax layer is added as the final output. Training of the local models was conducting using PyTorch library (version 1.3.1 including Torchvision version 0.4.2; Adam Paszke, Sam Gross, Soumith Chintala, and Gregory Chanan; 1601 Willow Rd, Menlo Park, CA 94025, USA), with CUDA support (version 9; Nvidia Corporation; 2788 San Tomas Expy, Santa Clara, CA 95051, USA), using GPU instances through Amazon Web Services (AWS).

#### Training procedure

Three different DAG topologies, namely (1) 5-node in 1-cluster, (2) 15-node in 1-cluster and (3) 15-node in 3-cluster (5 nodes each) as being described in more details in Supplementary Information section S1, were deployed. For non-medical dataset, a separate transfer dataset of 2000 cleansed images with equal class sizes, which is different from any training, validation and testing set, is used for decentralized training procedure. With more nodes involved in topologies (2) and (3), the number of images allocated at each node would be smaller (240 images per node in 15-node setting compared with 720 images per node in 5 node setting).

### Non-medical dataset composition

The dataset used for the following experiments includes images of cats and dogs, taken from ImageNet^35^, with the intention of using a binary classification problem as a known, solvable problem in which to trial the novel Decentralized AI Training technique. 4500 images (2250 cats and 2250 dogs) were used for training/validation sets while 4501 images (2253 cats and 2248 dogs) were used as the test set. The training/validation was shuffled and split 80/20, with 3600 images in the training set and 900 images in the validation set. The validation set is presumed to be sharable between different nodes or otherwise it is assumed to be kept separated from any nodes’ data. These original datasets are considered cleansed since there are no images of cats labeled “dog” and vice versa. Without the introduction of noise into the training and validation datasets, a trained deep AI model would approach maximum accuracy on the test set. The noisy datasets would also help to leverage the problem complexity and to demonstrate the differences between the new decentralized training and a more conventional centralized training regime. Different models were tested on their ability to handle and overcome some levels of noise. The noisy datasets were created by converting 10% of “dog” labels to “cat” labels (class 0), and 50% of “cat” to “dog” labels (class 1). This results in the amount of noise appearing in “cat” and “dog” classes, respectively, to be 17% and 36%. The different noise levels for each class were intentional, creating an unbalanced class distribution and uneven noise levels between two classes. The test set was kept clean in order to reliably compare different AI models’ performance.

In the 5-node in 1-cluster scenario, the training dataset is equally split between each node (720 training images per node). The clean data at each node contains 360 of either “cat” or “dog” class. If noise is introduced, each node has 216 images of cats and 504 images of dogs. In the 15-node scenario, 240 images are available at each node with 72 images labelled as cat and 168 images labelled as dog for the case of noisy data. The number of images summed from all nodes remains 3600. The centralized models were trained and validated on the centralized set of 3600 training images and 900 validation images (clean or noisy) with multiple hyper-parameter and model architecture settings. The best model was selected, forming a baseline for later comparison with new decentralized model results.

The choice of transfer set was then investigated using a node’s data as a transfer set or by using a combination of multiple nodes’ data. The effect of the training duration (number of epochs) of a Student model at each node was studied by varying the number of epochs, which allows the determination of practical bounds on the total number of (node-level) ‘epochs’ to consider when finalizing the training process across multiple nodes using distillation training.

In another scenario, the 15-node was divided into three equal clusters using the clustering method described above. The trade-off between data transfer (network) cost, and model accuracy was explored, providing a guide as to how to optimize decentralized training for real-world experiments.

### Medical dataset composition

Table 4 presents the data allocation to each node from a multi-center clinical datasets.

**Table 4:** Embryo dataset broken down to clinic owners.

Clinic data allocation	Non-viable	Viable	Total
**Training and validation set**	1251	942	2193
Node1—Fertility Associates NZ (FANZ)	269	318	587
Node2—Institute for Reproductive Health (IRH)	197	217	414
Node3—Repromed Adelaide (REP)	615	174	789
Node4—Ovation Austin (OVA)	79	157	236
Node5—Midwest Fertility Specialists And San Antonio IVF (MISA)	91	76	167
Clean test set	272	641	913
Noisy blind test set	517	681	1198

The data sizes vary from 167 to 587 images across different nodes. The total number of images for the training set is 2193, in which a validation set was randomly drawn and accounts for 20% of the original training set. If a centralized model is deployed, all these per-node data will be placed collectively in a single server regardless of the clinic information. The model trained using this centralized dataset, validated on the validation set, will form the baseline results which will be used to compare with the decentralized model trained on 5-node clinical data.

The Noisy Blind Test Set contains inherent errors in the Non-Viable class. Embryos labeled as Non-Viable may be Viable but extraneous patient factors (e.g. severe endometriosis) result in the patient not becoming pregnant. The Noisy Blind Dataset consists of 1198 original images collected from the same clinics allocated to four nodes above, namely, FANZ, IRH, OVA and MISA (this is a combination of smaller datasets from two clinics), and from 5 other unseen clinics including Alpha Fertility (Alpha), Flinders Fertility Adelaide (Flinders), Institute for Reproductive Health (IRH), Oregon Reproductive Medicine (ORM), Safe Fertility and Washington University at St Louis (Washington). Therefore, Node3 (REP clinic) contributes only to the training set, and the noisy blind test set contains representatives from 9 clinics total. The clinically realistic (albeit noisy) blind test set allowed us to practically assess the AI models’ performance (accuracy and generalizability) within and between clinics.

A clean test set was also created using a novel data cleansing method (UDC)^41^, from the noisy blind test set. The clean test set includes 913 images in which the viable embryos remain almost the same as in the original noisy dataset while approximately a half of non-viable embryos were identified as mislabeled and removed. The cleansed test dataset provides an unbiased assessment of the AI model’s performance.

**Figure 6:**
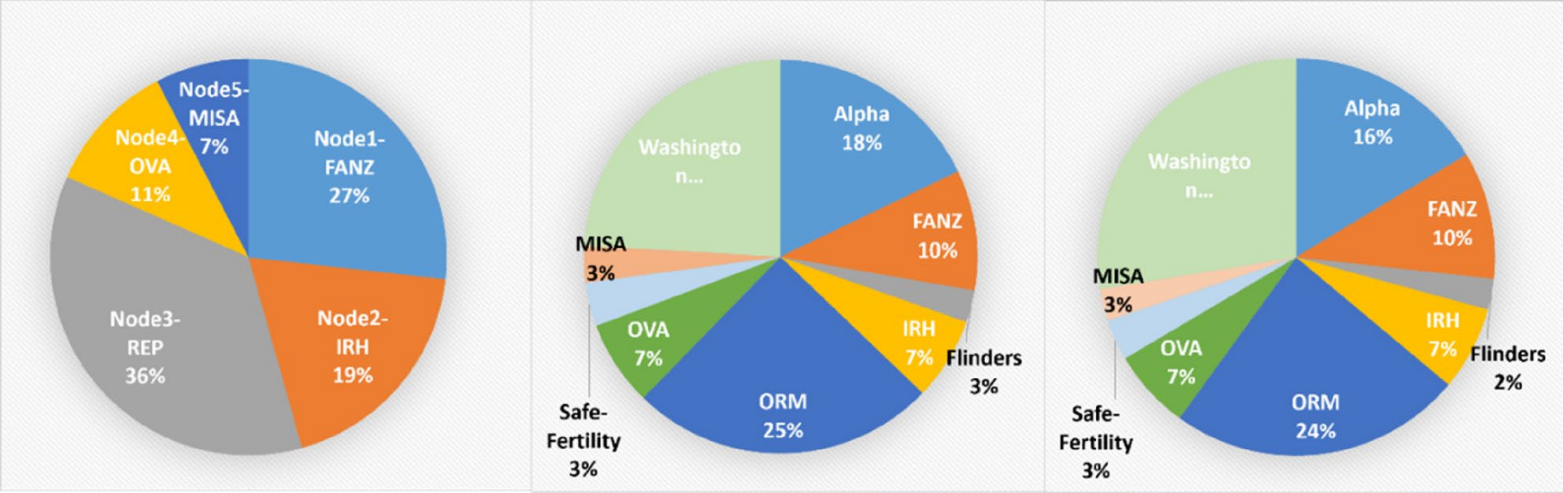
The clinics’ data size shown in percentages. Training dataset (left) with clinics’ data allocated to 5 nodes, cleansed test set (middle) and noisy blind test set (right).

Figure 6 presents the clinics’ data sizes in percentages. The circle graph on the left represents the training dataset with 5 node allocation, the graphs in the middle and on the right represent the clinic data distributions for the cleansed test set and the noisy blind test set, respectively. Generally, the image data provided from various clinics differ in image size/resolution, and in camera type and focal setting. The test sets are broader in the number of participated clinics and contain largely unequal-sized datasets contributed by those clinical centers. This diversity would pose significant challenges for a classifier in terms of generalizability across different clinics’ data.

### Ethics approval

This study was exempted from ethical review and approval, and from the requirement for informed consent due to the retrospective nature of the analyses, and de-identification of all data. Exemption was confirmed by Sterling Institutional Review Board (Sterling Independent Services, Inc.) committee ID #6467, for protocol ID LW-C-001A. This study was conducted according to the guidelines of the Declaration of Helsinki of 1975, as amended.

## Supplementary Information

Supplementary Information.

## Data Availability

Datasets generated during the current study are available from the corresponding author on reasonable request. Non-medical datasets are publicly available. Medical datasets are not publicly available due to data privacy restrictions.
