# Supplementary material for: A novel decentralized federated learning approach to train on globally distributed, poor quality, and protected private medical data
Source: Sci Rep. 2022 May 25;12:8888. doi: 10.1038/s41598-022-12833-x (PMC9133021; doi:10.1038/s41598-022-12833-x)
Supplement: Supplementary file 1 — Supplementary Information. [file 41598_2022_12833_MOESM1_ESM.docx]

## Distillation and Data Parallelism Training

One important benefit to using distillation is the extra control it provides in optimizing for the best ML solution to a decentralized data problem. Models trained on datasets within their own local nodes can be transferred to another node to become a Teacher model, at every epoch, or any larger number of epochs to reduce network transfer costs. Furthermore, the flexibility in transferring the model as a complete entity, only periodically, allows one to explore different strategies in which models are swapped from node to node, to optimize for the best generalized AI model, without violating privacy or confidentiality.

In training on distributed datasets, Data Parallelism strategy must be chosen in which the dataset is split into partitions. When data are allowed to be shared, typically each *training batch* is split into equal partitions equal to the number of nodes. For completely decentralized data where no data are allowed to be transferred, Data Parallelism commonly takes one of two forms:

a) Full Distributed Training, where model weight updates are sent to a Parameter server every batch and the Parameter server combines all weight updates into the ‘master’ model. This method is robust from a machine learning standpoint because the weights are transmitted periodically from every model to every other model, and a full model incorporating all weight updates into an optimization strategy (such as Stochastic Gradient Descent) can be created in one step. This method is extremely intensive in terms of network transfer costs and can typically be prohibitive for real-world problems^17^.

b) ‘Pattern’ or DAG-based Training, where there is no master node, and a communication strategy such as Ring Allreduce^28^, or other peer-to-peer network transfer is adopted. In this case, each worker independently averages model gradients it has received in parallel with its peer nodes.

## Three Level Weighting Loss Function for Decentralized Training

The process of weighting involves emphasizing the contribution of a subset of the data during model training. That is, rather than each variable in the dataset contributing equally to the model’s result, some of the data is adjusted to make a greater contribution than others.

**Sample/image level weighting:**

One might choose to emphasize training samples that were hard to classify and decreases the impact on easy ones. Mathematically, a scaling factor is added to the cross-entropy loss function. To identify the degree to which a sample is hard to detect, one might use the model’s prediction outcomes. For example, if the network output of a sample is 0.9, this could be seen as a relatively easy-to-detect sample if its class label is ‘1’, in a binary classification problem. On the other hand, the hard-to-detect samples would be the cases when the prediction scores deviate significantly from the target label.

For the sample/image weighting: the binary target label of an input sample *i_th_* is denoted as $y\in\{0,1\}$ specifying the ground-truth class. Assuming $p\in\left[ 0,1 \right]$being the model’s prediction score corresponding with the input *i_th_*. For notational convenience, we deﬁne *p_t_:*

$$p_{t}= \left\{ \begin{aligned} p if y=1 \\ 1-p otherwise \end{aligned} \right.$$

**Class level weighting:**

In the cases of unbalanced class distribution, one might want the model to focus more on the class that has fewer training samples, because the prediction model would then become biased on the dominant class samples and could be prone to assigning all training samples to the dominant class label. Based on the number of samples on each class, one can estimate the weighting ratio for each class that would help the model train in a balanced way across class samples.

We define the class weighting factor $\propto\in\left[ 0,1 \right]$ for class 1 and $1-\propto$ for class 0, $\propto_{t}$ is defined analogously to how $p_{t}$was deﬁned.

$$\propto_{t} =\left\{ \begin{aligned} \propto if y=1 \\ 1-\propto otherwise \end{aligned} \right.$$

**Distributed node level weighting:**

In the decentralized training, the amount of data available on each node possibly is significantly unbalanced. The decentralized model would be trained more frequently with a particularly small number of samples on nodes where a very small amount of data is available. This might result in an over-training, given those images. One might wish the model to emphasize the nodes where a large amount of data is available and vice versa.

Formally, given *k* nodes in decentralized training, each node holds a portion of training data. A ratio$\beta_{k}$is defined to determine how much the model emphasis on a specific node’s training data.

Finally, we define the three-level loss function, $\mathcal{L}\left( p_{t} \right)$for a model’ prediction outcome as follows:

$$\mathcal{L}\left( p_{t} \right)={-\propto}_{t}\beta_{k} \left( 1-p_{t} \right)^{\gamma}\log\left( p_{t} \right),$$

where $\gamma$ is used to control the influence level of per-sample weighting strategy.

## Decentralized Federated Learning Algorithm

We denote a training function $\mathcal{F}$ encapsulating the model architecture and parameter settings and procedures necessary to appropriately train a machine learning model. A trained model $\mathbb{M}$ is the result of the training function $\mathcal{F}$ on a dataset $\mathbb{D}$. This representation $\mathbb{M}^{\boldsymbol{(}\boldsymbol{i}^{\boldsymbol{'}}\boldsymbol{)}}\mathcal{\leftarrow F}\left\{ \mathbb{D,}S\mathcal{, L,}{e(\mathbb{M}}^{\left( i \right)},i\in\mathbb{N}_{1}^{n}) \right\}$ , where $\mathbb{N}_{1}^{n}$ is a set of positive numbers running from 1 to $n$, therefore means that the trained model $\mathbb{M}^{(i^{'})} would be obtained after model is trained$using the dataset $\mathbb{D}$, at location/server $S$, with the distillation loss function $\mathcal{L}$, based on the ensemble $e$ of the outcomes of $\mathbb{M}^{\left( i \right)},i\in\mathbb{N}_{1}^{n}$, where $i$ is a constant, and the ensemble $e$ simply takes the output of single model $\mathbb{M}^{\left( i \right)}, or {e(\mathbb{M}}^{\left( i \right)})=\mathbb{M}^{\left( i \right)}$. The training function $\mathcal{F}$ without $\mathcal{L,}e$ and $\mathbb{M}^{(i^{'})}$will become the standard Teacher model for each node, trained without the use of knowledge distillation. $\mathbb{M}^{(i^{'})}$can be incrementally trained multiple times with different $\mathcal{F}$ or particularly with different training sets or different Teachers.

To represent the actions regarding trained model files, we denote $\mathbb{M↠}S$being to copy and transfer a model $\mathbb{M}$ to the location/server $S$, $\mathbb{M}^{(a)}↞\mathbb{M}^{(b)}$being to create a new $\mathbb{M}^{(a)}$ by copying or cloning a version of $\mathbb{M}^{(b)}.$ The variables located within a bracket indicate the indices.

The decentralized Federated Learning algorithm is presented here in the form of a Main function which occasionally calls the child function named **LoopTrain**. The algorithm does not indicate where the parallelism could be taken place. All Student or Teacher models training without a dependency or sequential order can be deployed in parallel. The algorithm requires the following input.

## Algorithm description

The AI training process described herein in Algorithm 1 can be summarized as follows. If the total number of nodes is sufficiently large the decentralized training process becomes prohibitive in terms of compute power or cost, then a multiple level process can be carried out as illustrated in Figure 3. In this Figure, the 5-node (a) and 15-node (b) of a single cluster are shown in the form of a ring-like architecture. In some situations, the break-down to multiple level process is necessary which is illustrated in (c) with 3-cluster architecture, 5-node in each cluster. Generally, the *n* nodes are separated into *K* clusters so that there is a finite and smaller number of clusters than the total number of nodes ($K<n$). Separating the nodes into a cluster may be performed deterministically, for example based on geographic proximity, via random selection, or a hybrid approach. The nodes may be partitioned into large geographic regions, with random allocation of a node with a geographic region to multiple clusters (within a region).

Algorithm 1 can be extended to multiple level process in which a cluster may contain another set of child clusters. While the top/highest layer contains only the global center server $S^{c},$ the last layer contains edge/leaf nodes. For simplicity, Algorithm 1 assumes a 2-level process that is feasibly applicable in practice.

**Algorithm 1: Knowledge-based decentralized training**

**Input:** Data owner servers are divided into $K$ clusters_,_ $\mathbb{C}^{(k)}$ denotes $k$-th cluster in which the number of servers can be defined as $sizeof(\mathbb{C}^{(k)})$.

**Input:** Server $S^{(k,i)}$ is used for $i$-th data owner/server within $k$-th cluster.

**Input**: A data owner’s dataset $\mathbb{D}^{\left( k,i \right)}$ is located at $S^{(k,i)}$within $k$-th cluster, without external access. If a model $\mathbb{M}^{\boldsymbol{(}k\boldsymbol{,i)}}$ is created, it means the model is located at $S^{(k,i)}$, which is in server $i$-th and is in $k$-th cluster, and its training data is $\mathbb{D}^{\left( k,i \right)}$.

**Input**: For each cluster $\mathbb{C}^{(k)}$, there is a cluster-based central server $S^{(k)}$, each with its own (optional) local (transfer) dataset $\mathbb{D}^{\left( k \right)}$.

**Input**: A single global central server $S^{c}$, with its own (optional) local (transfer) dataset $\mathbb{D}^{c}$, the $S^{c}$ is directly connected with all $S^{(k)}$.

**Input**: Distillation loss function $\mathcal{L}$.

**Input**: $T$ is number of times around the nodes in a cluster that the model is sent for distillation training.

**Input**: $\boldsymbol{e}$ is the ensemble function that combines the outputs of Teacher models during distillation training.

| **1** | **FUNCTION: Main** ($K\mathbb{, C,}S,\mathbb{D}\mathcal{,L,}T,e)$ |
| --- | --- |
| **2** | **BEGIN** |
| **3** | **FOR** $k\boldsymbol{\in}\mathbb{N}_{1}^{K}$ **#** loop through each cluster $k$ |
| **4** | $n\boldsymbol{\leftarrow}\boldsymbol{sizeof}(\mathbb{C}^{(k)})$ |
| **5** | $\mathbb{M}^{(k,i)}\mathcal{\leftarrow F}\left\{ \mathbb{D}^{\left( k,i \right)},S^{\left( k,i \right)} \right\},i\in\mathbb{N}_{1}^{n}$ |
| **6** | **ENDFOR** |
| **7** | **FOR** $k\in\mathbb{N}_{1}^{K}$ |
| **8** | $n\boldsymbol{\leftarrow}\boldsymbol{sizeof}(\mathbb{C}^{(k)}$) |
| **9** | **FOR** $i\in\mathbb{N}_{1}^{n}$ |
| **10** | $\mathbb{M}^{I(k,i)}↞\mathbb{M}^{(k,i)}$ |
| **11** | $\mathbb{M}^{I(k,i)}=\boldsymbol{LoopTrain}\mathbf{(}\mathbb{M}^{I\left( k,i \right)}, S^{\left( k,j \right)},\mathbb{D}^{\left( k,j \right)}, \mathbb{M}^{\left( k,j \right)}, j\in\mathbb{N}_{1}^{n},T, use_{e}=false)$ |
| **12** | $\mathbb{M}^{I\left( k,i \right)}↠S^{\left( k \right)}$ |
| **12** | **ENDFOR** |
|  | *# At cluster level* |
| **13** | $\mathbb{M}^{k1}\mathcal{\leftarrow F}\left\{ \mathbb{D}^{\left( k \right)}, S^{\left( k \right)}\mathcal{, L,}{e\mathbb{(M}}^{I\left( k,i \right)},i\in\mathbb{N}_{1}^{n}) \right\}\Longleftrightarrow\mathbb{D}^{\left( k \right)}\neq\emptyset$ |
| **14** | $\mathbb{M}^{k2}\leftarrow$ ${e\mathbb{(M}}^{I\left( k,i \right)},i\in\mathbb{N}_{1}^{n})$ |
| **15** | $\mathbb{M}^{k3}=\boldsymbol{LoopTrain}(\emptyset, S^{\left( k,i \right)},\mathbb{D}^{\left( k,i \right)}, \mathbb{M}^{I\left( k,i \right)}, i\in\mathbb{N}_{1}^{n},T, use_{e}=true)$ |
| **16** | $\mathbb{M}^{b\left( k \right)}\mathbf{=}\boldsymbol{best}\boldsymbol{\{}\mathbb{M}^{k1},\mathbb{M}^{k2},\mathbb{M}^{k3}\boldsymbol{\}}$**,** given a validation set for model assessment. |
| **17** | $\mathbb{M}^{b(k)}↠S^{\left( c \right)}$ |
| **18** | **ENDFOR** |
|  | *# At global level, e.g. on a representative node* |
| **19** | $\mathbb{M}^{c1}\mathcal{\leftarrow F}\left\{ \mathbb{D}^{c}, S^{c}\mathcal{, L,}{e\mathbb{(M}}^{b\left( k \right)},k\in\mathbb{N}_{1}^{K}) \right\}\Longleftrightarrow\mathbb{D}^{c}\neq\emptyset$ |
| **20** | $\mathbb{M}^{c2}\mathbf{=}\boldsymbol{LoopTrain}(\emptyset, S^{\left( k \right)},\mathbb{D}^{\left( k \right)}, \mathbb{M}^{b\left( k \right)}, k\in\mathbb{N}_{1}^{K},T, use_{e}=true)\Longleftrightarrow\mathbb{D}^{\left( k \right)}\neq\emptyset$ |
| **21** | $\mathbb{M}^{c3}\boldsymbol{= \emptyset}$ |
| **22** | **FOR** $k\in\mathbb{N}_{1}^{K}$ |
| **23** | $n\boldsymbol{\leftarrow}\boldsymbol{sizeof}(\mathbb{C}^{(k)})$ |
| **24** | $\mathbb{M}^{c3}\mathbf{=}\boldsymbol{LoopTrain}\mathbf{(}\mathbb{M}^{c3}, S^{\left( k,i \right)},\mathbb{D}^{\left( k,i \right)}, \mathbb{M}^{I\left( k,i \right)}, i\in\mathbb{N}_{1}^{n}, T, use_{e}=true)$ |
| **25** | **ENDFOR** |
| **26** | $\mathbb{M}^{c}\mathbf{=}\boldsymbol{best}\boldsymbol{\{}\mathbb{M}^{c1},\mathbb{M}^{c2},\mathbb{M}^{c3}\boldsymbol{\}}$**,** given a validation set for model assessment. |
| **27** | **RETURN** $\mathbb{M}^{c}$ |
| **28** | **ENDFUNCTION** |
|  |  |
| **29** | **FUNCTION: LoopTrain(**$\mathbb{M}^{r},\left\{ S^{\left( i \right)} \right\},\left\{ \mathbb{D}^{\left( i \right)} \right\}, \left\{ \mathbb{M}^{\left( i \right)} \right\}, i\in\mathbb{N}_{1}^{n}, T, use_{e}=true)$ |
| **30** | **BEGIN** |
| **31** | **FOR** $r\in\mathbb{N}_{1}^{T}$ |
| **32** | **FOR** $i\in\mathbb{N}_{1}^{n}$ |
| **33** | $\mathbb{M}^{r}↠S^{\left( i \right)}\Longleftrightarrow\mathbb{M}^{r}\neq\emptyset$ |
| **34** | **IF** $use_{e}$ **THEN** |
| **35** | $\mathbb{M}^{(j)}↠S^{\left( i \right)}, j\in\mathbb{N}_{1}^{n}\Longleftrightarrow\mathbb{\nexists M}^{\left( j \right)} in S^{\left( i \right)}$ |
| **36** | $\mathbb{M}^{r}\mathcal{\leftarrow F}\left\{ \mathbb{D}^{\left( i \right)}, S^{\left( i \right)}\mathcal{, L,}{e\mathbb{(M}}^{\left( j \right)}), j\in\mathbb{N}_{1}^{n} \right\}$ |
| **37** | **ELSE** |
| **38** | $\mathbb{M}^{r}\mathcal{\leftarrow F}\left\{ \mathbb{D}^{\left( i \right)}, S^{\left( i \right)}\mathcal{, L,}\mathbb{M}^{\left( i \right)} \right\}$ |
| **39** | **ENDIF** |
| **40** | **ENDFOR** |
| **41** | **ENDFOR** |
| **42** | **RETURN** $\mathbb{M}^{r}$ |
| **43** | **ENDFUNCTION** |

First, each leaf node trains a local model $\mathbb{M}^{(k,i)}$using local dataset $S^{\left( k,i \right)}$(from line 3 to 6). This process can be made to run in parallel, given individual training processes being completely independent. Knowledge distillation is then used to train $n$ new models. Each new model $\mathbb{M}^{I(k,i)}$is initialized from the pre-trained local model $\mathbb{M}^{(k,i)}$ (line 10), and this new model then goes through a loop training process (line 11) which involving moving $\mathbb{M}^{I(k,i)}$ to other nodes cluster-wide, to distill knowledge from local pre-trained Teacher $\mathbb{M}^{I(k,i)}$. This process involves only one Teacher to one Student; hence no ensemble method is applicable. Once $\mathbb{M}^{I(k,i)}$complete its circuit to every node, a copy of it will be transferred to the direct parent cluster server $S^{\left( k \right)}$(line 12).

At the cluster level, three options are suggested: (1) if the transfer dataset $\mathbb{D}^{\left( k \right)}$is in cluster $k$, a new model $\mathbb{M}^{k1}$ is created and trained using $\mathbb{D}^{\left( k \right)}$ with the use of knowledge distillation from the ensemble of $\mathbb{M}^{I\left( k,i \right)}$ (line 13); (2) a new model $\mathbb{M}^{k2}$ can also be easily created from the ensemble of models, $\mathbb{M}^{I\left( k,i \right)}$(line 14); (3) and finally, a new model $\mathbb{M}^{k3}$ is initialized via a loop training process (line 15) which requires copying all models $\mathbb{M}^{I\left( k,i \right)}$ to each node $i$ within the $k$-th cluster, and to move $\mathbb{M}^{k3}$ to each leaf node to train on the local data and distill knowledge of the ensemble of models $\mathbb{M}^{I\left( k,i \right)}$. The next step would be to pick the best models among $\{\mathbb{M}^{k1},\mathbb{M}^{k2},\mathbb{M}^{k3}\}$, if more than one model is available, and if there exists a suitable validation set at $S^{\left( k \right)}$. The best model selected $\mathbb{M}^{b(k)}$ will then be transferred to global center server $S^{c}$(line 17).

At the global level, there are also three options: (1) if a transfer dataset $\mathbb{D}^{c}$is present, a new model $\mathbb{M}^{c1}$ is created and trained using $\mathbb{D}^{c}$ with the use of knowledge distillation from the ensemble of $\mathbb{M}^{b\left( k \right)}$ (line 19); (2) a new model $\mathbb{M}^{c2}$ can also be made from the ensemble of $\mathbb{M}^{b\left( k \right)}$(line 20); (3) and for the third option, a new model $\mathbb{M}^{c3}$ is initialized via a multi-node loop training process across every cluster (line 21 to 25). This does not require copying all $\mathbb{M}^{I\left( k,i \right)}$to each node $i$ within the $k$-th cluster, since this has been done previously at line 15. $\mathbb{M}^{c3}$is moved to each leaf node to train on the local data and distill knowledge of the ensemble of models $\mathbb{M}^{I\left( k,i \right)}$. The final step would be to pick the best models among $\{\mathbb{M}^{c1},\mathbb{M}^{c2},\mathbb{M}^{c3}\}$, if more than one model is available and if there exists a validation set at $S^{c}$. The best model selected $\mathbb{M}^{c}$ will be final model of this decentralized training process.

The loop training process is implemented separately in the function **LoopTrain**, which requires $\mathbb{M}^{r},\left\{ S^{\left( i \right)} \right\},\left\{ \mathbb{D}^{\left( i \right)} \right\}, \left\{ \mathbb{M}^{\left( i \right)} \right\}, i\in\mathbb{N}_{1}^{n}, T, use_{e}$ as its inputs. $\mathbb{M}^{r}$ can be initialized empty/null or can be a copy of a pre-trained model (line 33). In the loop, $\mathbb{M}^{r}$ will be iteratively trained given multiple datasets ${\mathbb{\{D}}^{\left( i \right)}\}$ located at server {$S^{\left( i \right)}\},$respectively. In total, $\mathbb{M}^{r}$will be trained $T$ journeys around the server set $\left\{ S^{\left( i \right)} \right\}, i\in\mathbb{N}_{1}^{n}$ (as shown in the two dependent loops at line 31 and 32). If distillation is used here, all models $\left\{ \mathbb{M}^{\left( i \right)} \right\}, i\in\mathbb{N}_{1}^{n}$ will be made available at any server in $\left\{ S^{\left( i \right)} \right\}, i\in\mathbb{N}_{1}^{n}$ (line 35), and $\mathbb{M}^{r}$ will distil knowledge from the ensemble of all $\left\{ \mathbb{M}^{\left( i \right)} \right\}, i\in\mathbb{N}_{1}^{n}$ (line 36). Otherwise, $\mathbb{M}^{r}$ will distil knowledge from single Teacher model $\mathbb{M}^{\left( i \right)}$ (line 38).

Note that the presence of a transfer dataset at each cluster central server, or at the global central server, is optional, as in practice it may not be possible to reserve a separate dataset to be used as a transfer set at each node. Thus, these optional datasets are used if available to generate additional models that can be used to select the best cluster model $\mathbb{M}^{b(k)}$, or to select the best global model $\mathbb{M}^{c}$. If there is only one cluster, such as in the cases of (a) and (b) in Figure 3, the global level operation becomes redundant, since the model, $\mathbb{M}^{b(k)}$, is the final model one would obtain without any further implementation.

## DAG topologies for decentralized training

In this article, the following DAG topologies were utilized.

### 5 Nodes in 1 Cluster

This 5-node, 1-cluster setting is illustrated in Figure 1a. The training procedure includes following steps: (1) At each node, a Specialist model is trained with local data for 20 epochs; (2) A Generalist model is created at each node (after all the Specialists being trained), for simplicity the Generalist model is a copy of the local Specialist model; (3) The Generalist model is sent around to other nodes, and at each node it learns the local data and distills knowledge from the local Specialist as its Teacher.

### 15 Nodes in 1 Cluster

This clustering arrangement is illustrated in Figure 1b. Key differences between 5-node and 15-node architectures are as follows: (1) The dataset available at each node is much smaller since more nodes are deployed; (2) The Specialist and Generalist model at each node is exposed to fewer images which is deliberately chosen to be on the lower limit to assure a good Specialist model; (3) When training the final model, all 15 trained Generalist models are used for the distillation strategy, hence the training process would take longer, and more memory is required to load all these 15 trained models. Data transfer is more significant for the 15-node case; (4) The final model is retained at each node for a fewer number of epochs compared with the 5-node case since local data size is smaller.

### 15 Nodes in 3 Clusters (5 Nodes Each)

This clustering arrangement is illustrated in Figure 1c. The main difference between this 3-cluster configuration and the 15-node 1-cluster is as follows. A Generalist model is only trained within its own cluster container until the final decentralized model $\mathbb{M}^{c}$ is created. When $\mathbb{M}^{c}$ traverses all clusters, it has an opportunity to learn every node’s data globally which is not possible with cluster-level Generalist models. $\mathbb{M}^{c}$, however, only extract the knowledge from one cluster at a time (for scalability reasons, the number of nodes allocated in each cluster should be relatively small to reduce data transfer and computing costs). Within each cluster, $\mathbb{M}^{c}$ is trained in the same way as in the 5-node 1-cluster architecture.

In this scenario, $\mathbb{M}^{c}$ is unable to distill knowledge simultaneously from all trained Generalist models as in the case with 15-node 1-cluster configuration. As a result, the decentralized AI model using the 3-cluster setting suffers from a performance decrease, compared to the 1-cluster case.
